# Visceral Leishmaniasis and HIV Coinfection in the Mediterranean Region

**DOI:** 10.1371/journal.pntd.0003021

**Published:** 2014-08-21

**Authors:** Begoña Monge-Maillo, Francesca F. Norman, Israel Cruz, Jorge Alvar, Rogelio López-Vélez

**Affiliations:** 1 Tropical Medicine & Clinical Parasitology, Infectious Diseases Department, Ramón y Cajal Hospital, Madrid, Spain; 2 WHO Collaborating Centre for Leishmaniasis, Servicio de Parasitología, Centro Nacional de Microbiología, Instituto de Salud Carlos III, Majadahonda, Madrid, Spain; 3 Visceral Leishmaniasis Program, Drugs for Neglected Diseases *initiative* (DND*i*), Geneva, Switzerland; National Institute of Allergy and Infectious Diseases, United States of America

## Abstract

Visceral leishmaniasis is hypoendemic in Mediterranean countries, where it is caused by the flagellate protozoan *Leishmania infantum*. VL cases in this area account for 5%–6% of the global burden. Cases of *Leishmania*/HIV coinfection have been reported in the Mediterranean region, mainly in France, Italy, Portugal, and Spain. Since highly active antiretroviral therapy was introduced in 1997, a marked decrease in the number of coinfected cases in this region has been reported. The development of new diagnostic methods to accurately identify level of parasitemia and the risk of relapse is one of the main challenges in improving the treatment of coinfected patients. Clinical trials in the Mediterranean region are needed to determine the most adequate therapeutic options for *Leishmania*/HIV patients as well as the indications and regimes for secondary prophylaxis. This article reviews the epidemiological, diagnostic, clinical, and therapeutic aspects of *Leishmania*/HIV coinfection in the Mediterranean region.

## Introduction

Visceral leishmaniasis (VL) is hypoendemic in the Mediterranean region, where it is caused by the protozoan *Leishmania infantum*. This parasite is transmitted by the bite of infected phlebotomine female sandflies of the *Phlebotomus* genus and is maintained in a zoonotic cycle, with dogs acting as the main reservoir [1].

Cases in the Mediterranean region only contribute to 5%–6% of the global burden of VL, with an estimated annual incidence of 1,200–2,000 cases [2], [3]. In Mediterranean countries, more than 27,000 new human immunodeficiency virus (HIV) infections were diagnosed in 2012, corresponding to rates of 6.6 per 100,000 people [4].

*Leishmania*/HIV coinfection was an emergent problem in the Mediterranean region during the 1990s, with cases occurring mainly in four countries: France, Italy, Portugal, and Spain. Currently, these countries still report most of the cases of coinfection in this region [2], [3], although the introduction of highly active antiretroviral therapy (HAART) in 1997 contributed to a marked decrease in cases of coinfection (from 1,440 cases during the period of 1990–1998 to 299 cases during 2001–2006, though this reduction was not so marked in Portugal) [3].

Clinical manifestations of leishmaniasis in HIV-positive patients in the Mediterranean region are not very different from those occurring in immunocompetent patients, although atypical symptoms and signs may occur [3].

Peripheral blood analysis by polymerase chain reaction (PCR) is a highly sensitive and specific tool to detect the parasite in coinfected patients using a less invasive approach [5]. More recently, the use of real-time PCR has been shown to be a suitable tool for monitoring parasite load during the follow-up of coinfected patients, helping to predict the risk of relapses after treatment [6], [7].

Specific *Leishmania*-HIV interactions at the cellular level might affect the course of infection for both pathogens. Visceral leishmaniasis seems to hamper the immunological competence of HIV-positive patients and heightens the increase in HIV load. Concomitant VL and HIV infection is characterized by significantly lower cure rates, higher rates of drug toxicity, and higher relapse and mortality rates when compared with HIV-negative VL patients.

The introduction of HAART in Europe has led to an improvement in the quality of life of coinfected patients, reducing the number of relapses as well as mortality, and has significantly decreased the number of new cases of coinfection. Nevertheless, VL relapses occur in patients on HAART despite increasing CD4+ counts and undetectable HIV loads [3], [8]. Moreover, VL also seems to hamper the immunological recovery of the HIV-positive patients treated with HAART [9].

The major clinical trials on treatment and secondary prophylaxis in coinfected patients have been carried out in southern European countries. For VL/HIV-coinfected patients in the Mediterranean area, amphotericin B (deoxycholate or the lipid or liposomal formulations) is the first-line therapeutic option, followed by maintenance therapy [10]–[12].

This article reviews the epidemiological, diagnostic, clinical, and therapeutic aspects of *Leishmania*/HIV coinfection in the Mediterranean region.

## Epidemiology of Visceral Leishmaniasis and HIV Coinfection in the Mediterranean Region

Isoenzyme typing of *L. infantum* strains isolated from HIV patients in the Mediterranean region shows a high level of polymorphism. Although most VL cases are caused by viscerotropic zymodemes, other dermotropic or dermo-viscerotropic strains and other new strains that have not been identified in HIV-negative patients are known to affect coinfected patients [13], [14].

*L. infantum* is transmitted by the bite of infected phlebotomine female sandflies of the *Phlebotomus* genus and maintained in a zoonotic cycle, with dogs acting as the main reservoir [1]. Data on *Leishmania* infections in other mammals are increasingly reported, although their role in transmission has yet to be elucidated [15]. Wild carnivores such as the wolf (*Canis lupus*), the red fox (*Vulpes vulpes*), the Egyptian mongoose (*Herpests ichneumon*), the genet (*Geneta geneta*), the pine marten (*Martes martes*), and the Iberian lynx (*Lynx pardinus*) have also been implicated in the maintenance of *L. infantum* transmission. Recently, infected Iberian hares (*Lepus granatensis*) have been associated with the ongoing outbreak in Spain [16].

Xenodiagnosis studies performed in the Mediterranean region have shown that *L. infantum* may develop in *Phlebotomus perniciosus* when it feeds on VL/HIV-coinfected patients [17]. Transmission may also occur through the sharing of contaminated syringes among intravenous drug users [18]. Furthermore, it has been shown that detection of parasitemia in peripheral blood samples by PCR is much more frequent among HIV/VL-coinfected patients than in non-HIV-infected patients with VL [19]. These data suggest that HIV/VL-coinfected patients may be more infectious than non-HIV-infected patients and that a simultaneous natural anthroponotic cycle could be considered in the epidemiology of VL due to *L. infantum* in HIV-coinfected patients [17]. In addition, *L. donovani*, which is mainly associated with anthroponotic transmission, has recently been identified as the etiological agent in cases of VL in Cyprus and Turkey [20].

Since the World Health Organization (WHO) discontinued its database in 2006, there is no centralized system updating data on VL/HIV. The last comprehensive information available was updated in 2007 during a WHO consultative meeting in Addis Ababa [21]. Since then, WHO has made efforts to set up a program in selected areas of the European region [22], [23]

Southern European countries are known hypoendemic areas for leishmaniasis, with a calculated incidence of 0.4 cases per 100,000 inhabitants per year in the case of Spain. Previously, two-thirds of patients were children, but after 1985 when the HIV pandemic started, up to 70% of the patients were adults, reflecting the pattern of HIV age risk groups [24]. The unexpectedly high rate of VL/HIV coinfection in Europe, mainly in Spain (where 80% of all cases notified to the WHO occurred [25]), indicate that HIV infection is a risk factor associated with VL [26]. In order to provide a population-based estimate of the burden of hospitalization caused by leishmaniasis and coexisting *Leishmania*/HIV, the Spanish Central Hospital Discharge Database (which includes data for >95% of all hospitalized patients in the whole country) was analyzed for the period 1997–2008. The prevalence of HIV-positive patients in the general hospitalized population was 0.58% [27], and the prevalence of HIV-positive patients among the 2,028 patients hospitalized with leishmaniasis was 37% [8]. Moreover, in an ongoing outbreak in southwest Madrid, a region with around 500,000 inhabitants, an incidence of 22.2 cases per 100,000 habitants was found, with 286 cutaneous leishmaniasis cases and 160 (35.9%) VL cases. Among the latter, 16 (10%) were VL/HIV-coinfected patients and 20 (12.5%) had other types of immunosuppression [28].

In Italy, the Campania region has the highest VL incidence, although HIV coinfection in this area is extremely rare. The regions contributing historically with most of the VL/HIV cases in this country were Sicily, Lombardy, and Latium, although leishmaniasis transmission is progressing to the more industrialized north (Valle d'Aosta, Lombardy, Venetto, etc.) where HIV is more prevalent. Currently, coinfected cases from these areas have already been reported, and careful surveillance is needed [29]. VL/HIV coinfection in the Maghreb seems to be a rare autochthonous problem [3], and this has been confirmed in a recent review [30].

HAART has contributed to the decrease in the incidence of VL/HIV cases in Europe, which has been observed since 1997 in all countries except Portugal, where, for unknown reasons, 107 coinfected cases among 173 VL patients were found during the period of 2000–2009 [31].

Visceral leishmaniasis and cutaneous leishmaniasis (CL) acquired in the Mediterranean area in countries such as Greece, France, Spain, Italy, Portugal, Turkey, and Croatia, which are considered premier tourist attractions, have been imported to northern European countries [32]–[34]. Moreover, imported cases to the Mediterranean region, mostly CL acquired by travelers to South and Central America [32], [35], have also been described.

## Clinical Manifestations of Visceral Leishmaniasis in HIV-Coinfected Patients

Typical manifestations of VL include fever, weight loss, hepatosplenomegaly, and pancytopenia resulting from replication of *Leishmania* amastigotes in macrophages mainly in the liver, spleen, and bone marrow.

Typical features such as splenomegaly may be absent in VL/HIV-coinfected patients [36], whereas atypical organ involvement, such as of the lungs or gastrointestinal system, may be found. Amyloid A (AA) amyloidosis leading to renal failure has been associated with chronic VL in HIV patients [37]–[39].

In patients infected with HIV, impaired immune function may favor the reactivation of latent *Leishmania* infections. Interactions between both pathogens in host cells may influence their expression and multiplication. Leishmaniasis can promote viral replication and enhance progression to AIDS. Other factors such as a shift towards a T helper 2 (Th2)-type specific response to *Leishmania* in patients with HIV-1-induced T lymphocyte dysfunction could also explain the severity of the infection and the atypical manifestations observed in some of these patients (in immunocompetent patients, T helper 1 (Th1) cellular immune responses to *Leishmania* have been associated with protection) [3], [19], [40].

Cutaneous involvement is infrequent in the context of *Leishmania*/HIV coinfection. In HIV patients, VL caused by *L. infantum* has been reported with simultaneous associated CL [41]. Other studies mention possible visceralisation of the infection from CL, as cutaneous infection predated visceral involvement [38]. Mucocutaneous lesions caused by *L. infantum* have also been described in HIV-positive patients [42]. *Leishmania* parasites have been detected in biopsies obtained from VL/HIV-coinfected patients that were performed to study other skin lesions, such as rheumatoid nodulosis and Kaposi's sarcoma [27], [43]. In some cases, the detection of *Leishmania* in cutaneous lesions led to the diagnosis of VL; however, the presence of the parasite in skin diseases in which it has no known etiological role may represent passive presence due to widespread dissemination in patients with compromised immune systems [37], [44]. Atypical disseminated cutaneous leishmaniasis (with diffuse, nonulcerated, maculopapular lesions) following visceral disease and post-kala-azar dermal leishmaniasis (PKDL) caused by *L. infantum* (an infrequent association) has also been described in HIV patients [45], [46]. Posterior uveitis has been reported in association with PKDL in a VL/HIV-coinfected patient on monthly liposomal amphotericin B prophylactic therapy [46].

A large proportion of the general population in the Mediterranean area have asymptomatic *L. infantum* infection, as detected by positive skin tests, serology, or peripheral blood PCR. Therefore, HIV-positive patients with asymptomatic *L. infantum* infection may also be expected. In fact, although patients infected with HIV have a high risk of developing symptomatic VL, several studies highlight the considerable proportion of these patients who may be asymptomatic carriers (cryptic infection) of *L. infantum* infection. In recent reports, around 10% and 17% of HIV+ individuals in southern France and Spain, respectively, were diagnosed with asymptomatic *Leishmania* infection by serology (western blot and immunofluorescent antibody test) [47], [48]. In another study in the south of Spain, *L. infantum* kinetoplast DNA was amplified from peripheral blood samples of around 30% of asymptomatic HIV-infected patients [49]. An association between high HIV viral load and high parasitemia has been reported, possibly related to an increased risk of progressing to symptomatic disease, although further studies are needed to establish this risk for symptomatic disease in these patients [19].

## Diagnostic Methods for Visceral Leishmaniasis in HIV-Coinfected Patients

The techniques for diagnosing *Leishmania* infection in HIV patients have not changed significantly in recent years, and the currently applied serological tests are not considered accurate methods for diagnosis because of limited sensitivity. According to a recent meta-analysis in Europe, immunoblotting showed the best performance, with 75%–91% sensitivity and 65%–94% specificity. Nonetheless, the available evidence indicates that serological tests should not be used to rule out VL in HIV-infected patients (Table 1) [50]. The combined use of the direct agglutination test (DAT) and the rK39-immunchromatographic test (rK39-ICT), both rapid and user-friendly methods, has been shown to be a suitable approach for VL diagnosis in Ethiopian HIV-positive patients, reaching a sensitivity of 98% [51]. The use of these two approaches for HIV-associated VL diagnosis in the Mediterranean region has not been properly evaluated in large series of VL/HIV-coinfected patients. A study in Italy using the rk39-ICT showed 100% sensitivity and specificity in 19 patients with confirmed VL, but only three of them were HIV positive [52]. Given the wide use and acceptance of DAT and rK39-ICT for VL diagnosis in other endemic areas [53], these two tests should be evaluated in the Mediterranean region to assess whether their use alone or in combination may improve the serodiagnosis of VL/HIV coinfection in this region.

**Table 1:** Estimated sensitivity and specificity of diagnostic tests based on antibody detection for VL in HIV-infected patients, using a random effects model and their respective 95% confidence intervals [50].

Diagnostic Test	Sensitivity (%)	95% CI	Specificity (%)	95% CI
Immunoblotting	84	75–91	82	65–94
DAT	81	61–95	90	66–100
ELISA	66	40–88	90	77–98
IFAT	51	43–58	93	81–99
PCR-blood	92	83–98	96	80–100
Antigen detection in urine	85–100	-	96–100	-

Data for antigen detection in urine [56], [55]. Abbreviations: CI, confidence interval; ELISA, enzyme-linked immunosorbent assay; IFAT, indirect fluorescent antibody test.

The detection of *Leishmania* antigen in urine using the latex agglutination test commercialized as KAtex initially appeared to be a promising, noninvasive tool for VL diagnosis and treatment follow-up. Its sensitivity in different studies in Europe, including both HIV-positive and HIV-negative patients, ranged from 69% to 100% [54], [55], and a positive result after treatment was strongly associated with relapse [54], [56], even though this was not confirmed in all studies [55]. However, decreased sensitivity and specificity in large diagnostic series [Israel Cruz, personal experience at WHO Collaborating Centre for Leishmaniasis, Madrid] has precluded a more widespread use of this test in the diagnosis of VL/HIV coinfection. Future research to improve the existing format would be necessary to obtain a useful noninvasive tool for diagnosis and treatment monitoring.

In recent years, peripheral blood-PCR analysis has been validated as a sensitive and specific tool to detect *Leishmania* parasites in coinfected patients using a minimally invasive technique [5], [50]. Therefore, whereas classical diagnostic methods, such as bone marrow aspirate culture and microscopy, are still in use, diagnosis in this region is mainly based on the combination of the molecular detection of parasite DNA in peripheral blood by PCR and serology (even considering the low sensitivity of the latter). Bone marrow aspirates are still a source for parasite detection by PCR because of increased sensitivity when compared with peripheral blood analysis [5]. Despite the widespread use of molecular diagnosis techniques for leishmaniasis in Europe and the various existing guidelines and algorithms for diagnosis in HIV patients, there is still a lack of consensus, with each laboratory using its own methodology. Thus, the development of common guidelines for diagnosis is needed.

The current challenge in the HAART era is to find accurate markers for prediction and detection of relapses, as these still occur despite the use of HAART. Some authors have proposed real-time PCR as a suitable tool for monitoring the parasite load during follow-up of coinfected patients and to predict the risk of relapses after treatment [6], [7], [57]. Parasite loads in VL relapses vary in different patients, as shown in several studies [6], [7], [57], [58], suggesting that other parameters should be taken into account. Cota et al. [59] identified some predictors of VL relapse in HIV-infected patients, such as the absence of an increase in CD4+ cells at follow-up, lack of secondary prophylaxis, and a previous history of VL relapse, and suggested that CD4+ counts below 100 cells/µL at the time of primary VL diagnosis may also be a predictive factor for VL relapse. These parameters and real-time PCR to assess parasite load are the tools currently available for monitoring VL in HIV-infected patients [6], [7], [57], [58].

New markers to assess cure and help predict relapses are still needed [1]. Pioneer studies by Moreno et al. [60] using lymphocyte blastogenesis assays and T cell subpopulation determination in a series of 17 VL HIV-infected Spanish patients indicated that the ability to mount and maintain a specific T cell response against *Leishmania* after treatment is necessary to decrease the risk of relapses. In fact, Bourgeois et al. [61], studying a cohort of 27 French VL HIV-infected patients, observed that a CD4 count <200 cells/µL was strongly associated with relapse episodes, regardless of use of HAART therapy and/or secondary prophylaxis against *Leishmania*.

Future research should focus on the assessment of cytokine profiles in larger series of patients. In an endemic area in Brazil, Costa et al. [62] found, in non-HIV patients, that following ex vivo stimulation with *Leishmania* antigens, the lymphocyte proliferative response and the interferon gamma (IFN-γ) production by lymphocyte cultures were higher in cured VL and asymptomatic *L. chagasi*–infected individuals than in active VL patients and healthy subjects from the same area. This would indicate a protective immune status. Cytokine release assays after ex vivo stimulation of peripheral blood cells are currently an important area of research in epidemiological studies assessing *Leishmania* exposure [63]–[65], as well as in studies aiming to define the immunological profile of VL patients and immune individuals [66]. A later study in India by Gidwani et al. [64] found that patients with active VL also presented a positive IFN-γ response after stimulation with *Leishmania* antigens. This surprising result indicated that the assessment of IFN-γ alone would not give an accurate clue to identify immune individuals. In the same endemic area, Singh et al. [66] used the same approach to assess IFN-γ, tumor necrosis factor alpha (TNF-α), and interleukin-10 (IL-10) cytokines and indicated that IL-10 was only released by stimulated peripheral blood cells of active VL patients. Thus, the simultaneous assessment of IL-10 and IFN-γ could reflect more aptly the immunological response that could distinguish between those with active disease and cured or subclinically infected, immune individuals. These studies could also be applied to the follow-up of HIV-infected patients with VL in the Mediterranean Basin, but data are currently lacking in coinfected patients.

Although a clinical practice guideline for VL/HIV diagnosis has been published by the World Health Organization [1], there are no specific guidelines for the European region. Therefore, the methods for diagnosis and follow-up (to determine cure and to predict relapses) vary considerably but are mainly based on culture of buffy coat from peripheral blood, blood PCR, and bone marrow aspirate microscopy and culture and/or bone marrow PCR.

## Therapeutic and Prophylactic Strategies for Visceral Leishmaniasis in HIV-Coinfected Patients

The management of VL/HIV-coinfected patients may be complex. These patients generally have lower cure rates and higher mortality rates than HIV-negative patients, more treatment failures, toxicity and resistance to pentavalent antimonial compounds [67], and more relapses, especially if CD4+ counts are <200 cel/µl. These factors reduce the pharmacological options because the response to treatment also decreases after multiple relapses [59]. A few clinical trials regarding the efficacy of treatment in coinfected patients have been published. To date, there is no general consensus on the drug of choice, dose, or duration or on the efficacy of combined therapies and maintenance therapy as secondary prophylaxis.

Based on published studies on VL/HIV-coinfected patients in the Mediterranean area, amphotericin B (deoxycholate, the lipid formulation, or the liposomal formulation) would be the first-line therapeutic option. Although the only clinical trials performed in the Mediterranean region have been performed with amphotericin B deoxycholate (AB) and amphotericin B lipid complex (ABLC), currently the WHO and other international guidelines recommend liposomal amphotericin B (LAB) as the first option because of its safety profile and cure rates as reported in studies performed in other geographical areas with other *Leishmania* species [68], [69]. Antimonials have been compared with AB and ABLC and have similar cure rates but more severe toxicity, so they should be considered only as second-line drugs [10], [11], [67], [70].

Experience with miltefosine is limited, and although initial cure rates were favorable, almost all patients relapsed [71]. Experience with other drugs such as pentamidine or paromomycin is limited to clinical cases, and these are mainly administrated in combination with other drugs [72], [73].

Many experts advocate for combined therapy among VL/HIV-coinfected patients in order to increase efficacy, especially in those patients with multiple relapses, and to decrease the emergence of resistant parasites. However, data to assess the efficacy of antileishmanial combination therapy in VL/HIV patients are insufficient, and no clinical trials have been performed in the Mediterranean area [3]. Only isolated cases of combination regimens with pentamidine and fluconazol, miltefosine and sodium stibogluconate [72], [74], allopurionol and meglumine antimoniate [75], or LAB and the human recombinant granulocyte-macrophage colony-stimulating factor (rhuGM-CSF) colony growth factor [76] have been published with good results, but they are insufficient to establish firm recommendations. Future research should probably be focused on regimens based on the combination of LAB and other second-line drugs such as miltefosine, paromomycin, or pentamidine. In fact, the Drugs for Neglected Diseases Initiative (DNDi) is performing a randomized clinical trial comparing LAB alone versus LAB in combination with miltefosine in Ethiopian VL/HIV-coinfected patients, but this has just started, and no data are available yet. Treatment recommendations for VL/HIV-coinfected patients in the Mediterranean and grades of evidence based on the Infectious Diseases Society of America (IDSA) grade classification [77], [78] are summarized in Table 2.

**Table 2:** Therapy for visceral leishmaniasis in HIV-coinfected patients in the Mediterranean area.

Treatment	Grade for VL/HIV in the Mediterranean Basin Caused by *L. infantum*
Amphotericin B deoxycholate 20 mg/kg as 0.7 mg/kg/day IV for 28 days.	BI
Amphotericin B lipid complex total dose 30 mg/kg as 3 mg/kg/day IV for 10 days	BI
Liposomal amphotericin B total dose of 50 (40–60) mg/kg as 4 mg/kg/day IV on days 1–5, 10, 17, 14, 31, and 38	BIII
Meglumine antimoniate (IM or IV): 20 mg Sb^v+^/kg/d (without upper limit of 850 mg/d) for 28 d	CI
Miltefosine: 100–150 mg/day po for 28 days	CIII
Combination therapy: Liposomal amphotericin B+paromomycin or miltefosine	No data

Evidence-based recommendation. **Strength of recommendation:** **A** = Good evidence to support a recommendation for use; **B** = Moderate evidence to support a recommendation for use; **C** = Poor evidence to support a recommendation; **D** = Moderate evidence to support a recommendation against use; **E** = Good evidence to support a recommendation against use.

**Quality of evidence**: **I** = Evidence from one or more randomized clinical trials; **II** = Evidence from one or more well-designed clinical trials, without randomization; from cohort or case-controlled analytic studies (preferably from >1 center); from multiple time series; or from dramatic results from uncontrolled experiments; **III** = Evidence from opinions of respected authorities, based on clinical experience, descriptive studies, or reports of expert committees [77], [78]. Abbreviations: IM, intramuscular; IV, intravenous; po, per os.

Most available data on secondary prophylaxis after a treated episode of VL in HIV-infected patients have been reported from Europe, where zoonotic transmission of *L. infantum* occurs. Such studies are mostly based on secondary prophylaxis with ABLC [79] and LAB [80]. Other drugs used for secondary prophylaxis but with less supporting evidence are pentavalent antimonials [81], pentamidine [82]–[84], miltefosine [85], azole drugs [86], [87], and allopurinol, alone or in combination (Table 3) [88]–[90].

**Table 3:** Studies on secondary prophylaxis regimens performed in the Mediterranean region for visceral leishmaniasis in HIV+ patients.

	Reference	Number of Patients, Country	Drug Regimen	Outcome
Amphotericin B Lipid Formulations	López-Vélez R et al. [79]	N = 17, Spain	Group 1 (N = 8): Amphotericin B lipid complex (IV) 3 mg/kg/day every 21 days. Group 2 (N = 9): No treatment.	Follow-up for 12 months. 50% and 22.2% relapse-free, respectively.
	Molina I et al. [80]	N = 17, Spain	All patients received for VL episode liposomal amphotericin B 4 mg/kg/day (IV) for 5 consecutive days followed by one dose per week for 5 weeks.	Median follow-up time was 14 months (range 5–44 months). Calculated probability of being relapse-free was 89.7%, 79.1%, and 55.9% at 6, 12, and 36 months follow-up, respectively.
Miltefosine	Marques N et al. [85]	N = 5, Portugal	All patients received miltefosine 50 mg (po) 3 times a week.	Treatment was performed until >250 CD4/mm^3^ and for a minimum of 12 months (12–24). Three patients were followed up after miltefosine was discontinued (8–28 months). All patients were relapse-free.
Pentavalent Antimonials	Ribera E et al. [81]	N = 46, Spain	Group 1 (N = 20): No treatment. Group 2 (N = 9): Allopurinol 300 mg/8 h (po). Group 3 (N = 17): Pentavalent antimonials 850 mg (parenteral) once a month.	Patients were followed up until relapse. 35%, 44%, and 82% were relapse-free, respectively.
Pentamidine	Pérez Molina JA et al. [84]	N = 6, Spain	Group 1 (N = 3): Pentamidine isethionate (IV) 4 mg/kg every 2 weeks. Group 2 (N = 3): Pentamidine isethionate (IV) every 4 weeks.	Average follow-up was 8 months (3–12 months). One relapse in group 2, 7 weeks after pentamidine was stopped.
	Patel TA et al. [83]	N = 4, United Kingdom	Patient 1 & 2: pentamidine (IV) 6 mg/kg every 3 weeks. Patient 3 & 4: pentamidine (IV) 6 mg/kg fortnightly.	Follow-up from 5 months to 4 years. All relapse-free during follow-up.
Azoles	Angarano G et al. [86]	N = 5, Italy	All received itraconazole 600 mg/day (po) in two doses	Maintenance treatment from 6 to 24 months. All relapse-free during follow-up except for one.
Combined Therapies	Barragán P et al. [90]	N = 1, Spain	Itraconazole 400 mg/day (po) plus miltefosine 150 mg/day with 1 month on and 2 months off schedule.	19 months of treatment relapse- free.
	Torrus D et al. [89]	N = 2, Spain	Fluconazol 200 mg (po) plus allopurinol 300 (po) daily.	Follow-up at 9 and 11 months. Both patients relapse-free

Most experts judge that once patients have recovered their immune function with HAART and the VL is quiescent, prophylaxis could be stopped when CD4+ count is maintained at >200 cells/µL for more than 6 months [91], [92].

## The Impact of Highly Active Antiretroviral Therapy on Visceral Leishmaniasis in HIV-Coinfected Patients

The HAART-induced reconstitution of cellular immunity seems to be the main determinant in reducing opportunistic infections in HIV-positive individuals. However, several studies have shown that HIV-1 protease inhibitors (PI) may exert antiparasitic effects directly. This could be explained by the fact that proteases of certain parasites could be an unspecific target for HIV-1 PI [93].

HAART in VL/HIV-coinfected patients in the Mediterranean area has achieved a 50%–60% reduction in the VL incidence, with higher survival rates and a reduction in relapse rates [9], [25]. However, two novel entities have been described in association with VL/HIV after the introduction of HAART in the Mediterranean Basin. HAART may produce an immune reconstitution inflammatory syndrome (IRIS) with AIDS-associated leishmaniasis resembling PKDL due to *L. infantum* even many months or years after the diagnosis of VL [94], [95]. HAART may also lead to asymptomatic carriers [6], [19], [49], and these may pose a risk for transmission in areas where the sandfly vector is present [7].

## Future Considerations

Currently, leishmaniasis is spreading northwards in endemic regions, outbreaks are occurring in endemic areas, and foci of the disease are appearing in previously nonendemic European countries [96], [97]. Thus, awareness about leishmaniasis should be increased among health professionals [98]. Moreover, regional and international VL control programs (including surveillance) within these regions are needed.

For *Leishmania*/HIV-coinfected patients, serological tests have a low sensitivity, and cross-reactions are possible [99]. Improved antigen detection tests are of paramount importance for use as tests of cure and to monitor relapses [56]. Considering the unfavourable prognosis of coinfection and the high risk of relapses, development of (bio) markers would be crucial in order to link the clinical outcome and the parasitological status and establish better criteria of cure.

Recurrent relapses may select resistant clones that could contribute to the spread of drug resistance, especially in anthroponotic leishmaniasis settings [3]. Research should focus on the use of combination therapy in these patients to reduce the number of relapses, prevent resistance, and reduce toxicity, avoiding the use of the highly cardiotoxic pentavalent antimonials.

There are no well-established criteria on which drug should be used for secondary prophylaxis, and clinical trials in this regard are necessary. Secondary prophylaxis with a drug different than that used for treating the primary VL attack would be recommended to minimize the risk of resistance spreading. However, these studies may be difficult to perform given the low prevalence of coinfection in Europe, and some conclusions may have to be extrapolated from data obtained in other settings [38].

Specific preventive measures for VL among HIV patients in the Mediterranean region have not been addressed. However, based on the hypothesis of possible anthroponotic artificial transmission by contaminated syringes, preventive measures focused on avoiding needle sharing among intravenous drug users may reduce transmission in the Mediterranean region [18]. Systematic screening and primary prophylaxis for VL among HIV patients as a preventive measure is not recommended in international guidelines. However, this probably merits further exploration in VL-endemic areas [100].

**Key Learning Points**

- VL is endemic in the Mediterranean region, where the causative agent is the protozoan parasite *L. infantum*. Cases in the region only contribute to 5%–6% of the global burden of VL, with an estimated annual incidence of 1,200–2,000 cases.
- The synergy between HIV and *Leishmania* can lead to protean manifestations that may hinder the clinical recognition of VL.
- PCR is a highly sensitive and specific noninvasive tool that may be used to detect the parasite in HIV-coinfected patients in the Mediterranean region. Real-time PCR could be a suitable tool for monitoring the parasite load during follow-up of coinfected patients and for predicting the risk of relapses.
- Treatment of VL/HIV-coinfected patients is less effective than in HIV-negative patients, and relapses are much more frequent. Clinical trials on the efficacy of treatment in patients coinfected by VL/HIV are scarce. Amphotericin B and lipid formulations are the most frequently used drugs in Mediterranean countries despite the low evidence of efficacy. Secondary prophylaxis seems to reduce relapses rates; however, there is little evidence regarding the type of drugs, doses, and duration of maintenance therapy.
- HAART for HIV patients in the Mediterranean region has reduced the incidence of new VL cases, increased survival rates, and reduced relapse rates.

**Top Five Papers in the Field**

- Alvar J, Aparicio P, Aseffa A, Den Boer M, Cañavate C, et al. (2008) The Relationship between Leishmaniasis and AIDS: The Second 10 Years. Clin Microbiol Rev 21: 334–359.
- Cota GF, de Sousa MR, Rabello A (2011) Predictors of Visceral Leishmaniasis Relapse in HIV-Infected Patients: A Systematic Review. PLoS Negl Trop Dis 5: e1153.
- Cota GF, de Sousa MR, Demarqui FN, Rabello A (2012) The Diagnostic Accuracy of Serologic and Molecular Methods for Detecting Visceral Leishmaniasis in HIV-Infected patients: Meta-Analysis. PLoS Negl Trop Dis 6: e1665.
- Laguna F, López-Vélez R, Pulido F, Salas A, Torre-Cisneros J, et al. (1999) Treatment of Visceral Leishmaniasis in HIV-Infected Patients: A Randomized Trial Comparing Meglumine Antimoniate with Amphotericin B. Spanish HIV-Leishmania Study Group. AIDS 13: 1063–1069.
- Laguna F, Videla S, Jiménez-Mejías ME, Sirera G, Torre-Cisneros J, et al. (2003) Amphotericin B Lipid Complex versus Meglumine Antimoniate in the Treatment of Visceral Leishmaniasis in Patients Infected with HIV: A Randomized Pilot Study. J Antimicrob Chemother 52: 464–468.

## References

[1] World Health Organization (WHO) (2010) Control of the leishmaniases: Report of a meeting of the WHO Expert Committee on the Control of Leishmaniases, 22–26 March 2010; Geneva. WHO technical report series, no. .949. Available: http://whqlibdoc.who.int/trs/WHO_TRS_949_eng.pdf. Accessed 10 June 2014.

[2] Alvar J, Velez ID, Bern C, Herrero M, Desjeux P, et al (2012) WHO Leishmaniasis Control Team: Leishmaniasis Worldwide and Global Estimates of Its Incidence. PLoS ONE 7: e35671. 22693548 10.1371/journal.pone.0035671 PMC3365071

[3] Alvar J, Aparicio P, Aseffa A, Den Boer M, Cañavate C, et al (2008) The relationship between leishmaniasis and AIDS: the second 10 years. Clin Microbiol Rev 21: 334–359. 18400800 10.1128/CMR.00061-07 PMC2292576

[4] European Centre for Diseases Control, WHO Regional Office for Europe (2012) Surveillance Report. HIV/AIDS Surveillance in Europe 2012. Available: http://www.euro.who.int/__data/assets/pdf_file/0018/235440/e96953.pdf. Accessed 10 June 2014

[5] Antinori S, Calattini S, Longhi E, Bestetti G, Piolini R, et al (2007) Clinical use of polymerase chain reaction performed on peripheral blood and bone marrow samples for the diagnosis and monitoring of visceral leishmaniasis in HIV-infected and HIV-uninfected patients: a single-center, 8-year experience in Italy and review of the literature. Clin Infect Dis 44: 1602–1610. 17516404 10.1086/518167

[6] Bourgeois N, Bastien P, Reynes J, Makinson A, Rouanet I, et al (2010) ‘Active chronic visceral leishmaniasis’ in HIV-1-infected patients demonstrated by biological and clinical long-term follow-up of 10 patients. HIV Med 11: 670–673. 20500233 10.1111/j.1468-1293.2010.00846.x

[7] Molina I, Fisa R, Riera C, Falco V, Elizalde A, et al (2013) Ultrasensitive Real-Time PCR for the Clinical Management of Visceral Leishmaniasis in HIV-Infected Patients. Am J Trop Med Hyg 89: 105–110. 23629932 10.4269/ajtmh.12-0527 PMC3748463

[8] Gil-Prieto R, Walter S, Alvar J, de Miguel AG (2011) Epidemiology of leishmaniasis in Spain based on hospitalization records (1997–2008). Am J Trop Med Hyg 85: 820–825. 22049034 10.4269/ajtmh.2011.11-0310 PMC3205626

[9] Lopez-Velez R (2003) The impact of highly active antiretroviral therapy (HAART) on visceral leishmaniasis in Spanish patients who are co-infected with HIV. Ann Trop Med Parasitol 97 Suppl 1: 143–147. 14678641 10.1179/000349803225002615

[10] Laguna F, Lopez-Velez R, Pulido F, Salas A, Torre-Cisneros J, et al (1999) Treatment of visceral leishmaniasis in HIV-infected patients: a randomized trial comparing meglumine antimoniate with amphotericin B. Spanish HIV-Leishmania Study Group. AIDS 13: 1063–1069. 10397536 10.1097/00002030-199906180-00009

[11] Laguna F, Videla S, Jimenez-Mejias ME, Sirera G, Torre-Cisneros J, et al (2003) Amphotericin B lipid complex versus meglumine antimoniate in the treatment of visceral leishmaniasis in patients infected with HIV: a randomized pilot study. J Antimicrob Chemother 52: 464–468. 12888588 10.1093/jac/dkg356

[12] Monge-Maillo B, Lopez-Velez R (2013) Therapeutic Options for Visceral Leishmaniasis. Drugs 73: 1863–1888. 24170666 10.1007/s40265-013-0133-0 PMC3837193

[13] Gramiccia M, Scalone A, Di Muccio T, Orsini S, Fiorentino E, et al (2013) The burden of visceral leishmaniasis in Italy from 1982 to 2012: a retrospective analysis of the multi-annual epidemic that occurred from 1989 to 2009. Euro Surveill 18: 20535. 23929120

[14] Chicharro C, Jimenez MI, Alvar J (2003) Iso-enzymatic variability of *Leishmania infantum* in Spain. Ann Trop Med Parasitol 97 Suppl 1: : 57–64. 14678633 10.1179/000349803225002534

[15] Antoniou M, Gramiccia M, Molina R, Dvorak V, Volf P (2013) The role of indigenous phlebotomine sandflies and mammals in the spreading of leishmaniasis agents in the Mediterranean region. Euro Surveill 18: 20540. 23929183 10.2807/1560-7917.es2013.18.30.20540

[16] Molina R, Jimenez MI, Cruz I, Iriso A, Martin-Martin I, et al (2012) The hare (*Lepus granatensis*) as potential sylvatic reservoir of *Leishmania infantum* in Spain. Vet Parasitol 190: 268–271. 22677135 10.1016/j.vetpar.2012.05.006

[17] Molina R, Lohse JM, Pulido F, Laguna F, Lopez-Velez R, et al (1999) Infection of sand flies by humans coinfected with *Leishmania infantum* and human immunodeficiency virus. Am J Trop Med Hyg 60: 51–53. 9988321 10.4269/ajtmh.1999.60.51

[18] Cruz I, Morales MA, Noguer I, Rodriguez A, Alvar J (2002) Leishmania in discarded syringes from intravenous drug users. Lancet 359: 1124–1125. 11943264 10.1016/s0140-6736(02)08160-6

[19] Colomba C, Saporito L, Vitale F, Reale S, Vitale G, et al (2009) Cryptic *Leishmania infantum* infection in Italian HIV infected patients. BMC Infect Dis 9: 199. 20003257 10.1186/1471-2334-9-199 PMC2797011

[20] Gouzelou E, Haralambous C, Amro A, Mentis A, Pratlong F, et al (2012) Multilocus microsatellite typing (MLMT) of strains from Turkey and Cyprus reveals a novel monophyletic *L. donovani* sensu lato group. PLoS Negl Trop Dis 6: e1507. 22348162 10.1371/journal.pntd.0001507 PMC3279343

[21] World Health Organizationn (2007) Fifth Consultative Meeting on Leishmania/HIV Co-infection; 20–22 March 2007; Addis Ababa, Ethiopia. WHO/CDS/NTD/IDM/2007.5. Available: http://www.who.int/leishmaniasis/resources/Leishmaniasis_hiv_coinfection5.pdf. Accessed 18 July 2014.

[22] World Health Organization (2009) Leishmaniasis in the European Region, a WHO consultative intercountry meeting; 17–19 November 2009; Istanbul, Turkey.

[23] WHO Regional Office for Europe (2013) Outlining a strategic framework on Leishmaniasis control—Report of a WHO meeting, Tbilisi, Georgia; 16–18 April 2013. Copenhagen, WHO Regional Office for Europe. Available: http://www.euro.who.int/__data/assets/pdf_file/0008/239426/OUTLINING-A-STRATEGIC-FRAMEWORK-ON-LEISHMANIASIS-CONTROL.pdf. Accessed 18 July 2014.

[24] Alvar J (1994) Leishmaniasis and AIDS co-infection: the Spanish example. Parasitol Today 10: 160–163. 15275487 10.1016/0169-4758(94)90270-4

[25] Desjeux P, Alvar J (2003) Leishmania/HIV co-infections: epidemiology in Europe. Ann Trop Med Parasitol 97 Suppl 1: : 3–15. 14678629 10.1179/000349803225002499

[26] Pintado V, Martin-Rabadan P, Rivera ML, Moreno S, Bouza E (2001) Visceral leishmaniasis in human immunodeficiency virus (HIV)-infected and non-HIV-infected patients. A comparative study. Medicine (Baltimore) 80: 54–73. 11204503 10.1097/00005792-200101000-00006

[27] Ministry of Health, Social Services and Equality (2010) Hospital Discharge Records in the National Health System (CMBD). CMBD date 2010. Available: http://www.msc.es/en/estadEstudios/estadisticas/cmbdhome.htm. Accessed 28 November 2013.

[28] Arce A, Estirado A, Ordobas M, Sevilla S, Garcia N, et al (2013) Re-emergence of leishmaniasis in Spain: community outbreak in Madrid, Spain, 2009 to 2012. Euro Surveill 18: 20546. 23929177 10.2807/1560-7917.es2013.18.30.20546

[29] Gramiccia M, Scalone A, Di Muccio T, Orsini S, Fiorentino E, et al (2013) The burden of visceral leishmaniasis in Italy from 1982 to 2012: a retrospective analysis of the multi-annual epidemic that occurred from 1989 to 2009. Euro Surveill 18: 20535. 23929120

[30] Chaara D, Haouas N, Dedet JP, Babba H, Pratlong F (2014) Leishmaniases in Maghreb: An endemic neglected disease. Acta Trop 132C: 80–93. 10.1016/j.actatropica.2013.12.018 24412727

[31] Campino L, Maia C (2010) Epidemiology of leishmaniases in Portugal. Acta Med Port 23: 859–864. 21144327

[32] Neghina R, Neghina AM (2010) Leishmaniasis, a global concern for travel medicine. Scand J Infect Dis 42: 563–570. 20438287 10.3109/00365541003789473

[33] Herremans T, Pinelli E, Casparie M, Nozari N, Roelfsema J, et al (2010) Increase of imported Leishmaniasis in the Netherlands: a twelve year overview (1996–2007). Int Health 2: 42–46. 24037049 10.1016/j.inhe.2009.12.005

[34] Ehehalt U, Schunk M, Jensenius M, van Genderen PJ, Gkrania-Klotsas E, et al (2013) Leishmaniasis acquired by travellers to endemic regions in Europe - A EuroTravNet multi-centre study. Travel Med Infect Dis 12: 167–172. 24388687 10.1016/j.tmaid.2013.12.003

[35] Pavli A, Maltezou HC (2010) Leishmaniasis, an emerging infection in travelers. Int J Infect Dis 14: e1032–e1039. 20952234 10.1016/j.ijid.2010.06.019

[36] Mondain-Miton V, Toussaint-Gari M, Hofman P, Marty P, Carles M, et al (1995) Atypical leishmaniasis in a patient infected with human immunodeficiency virus. Clin Infect Dis 21: 663–665. 8527563 10.1093/clinids/21.3.663

[37] Romeu J, Milla F, Batlle M, Sirera G, Ferrandiz C, et al (1991) Visceral leishmaniasis involving lung and a cutaneous Kaposi's sarcoma lesion. AIDS 5: 1272. 10.1097/00002030-199110000-00025 1786160

[38] Lopez-Velez R, Perez-Molina JA, Guerrero A, Baquero F, Villarrubia J, et al (1998) Clinicoepidemiologic characteristics, prognostic factors, and survival analysis of patients coinfected with human immunodeficiency virus and Leishmania in an area of Madrid, Spain. Am J Trop Med Hyg 58: 436–443. 9574788 10.4269/ajtmh.1998.58.436

[39] de Valliere S, Mary C, Joneberg JE, Rotman S, Bullani R, et al (2009) AA-amyloidosis caused by visceral leishmaniasis in a human immunodeficiency virus-infected patient. Am J Trop Med Hyg 81: 209–212. 19635871

[40] Cacopardo B, Nigro L, Preiser W, Fama A, Satariano MI, et al (1996) Prolonged Th2 cell activation and increased viral replication in HIV-Leishmania co-infected patients despite treatment. Trans R Soc Trop Med Hyg 90: 434–435. 8882199 10.1016/s0035-9203(96)90538-6

[41] Postigo C, Llamas R, Zarco C, Rubio R, Pulido F, et al (1997) Cutaneous lesions in patients with visceral leishmaniasis and HIV infection. J Infect 35: 265–268. 9459400 10.1016/s0163-4453(97)93080-2

[42] Aliaga L, Cobo F, Mediavilla JD, Bravo J, Osuna A, et al (2003) Localized mucosal leishmaniasis due to *Leishmania (Leishmania) infantum*: clinical and microbiologic findings in 31 patients. Medicine (Baltimore) 82: 147–158. 12792301 10.1097/01.md.0000076009.64510.b8

[43] Garcia-Rio I, Dauden E, Ballestero-Diez M, Fraga J, Garcia-Diez A (2010) Leishmaniasis and rheumatoid nodulosis in a patient with HIV infection. Actas Dermosifiliogr 101: 164–167. 20223159

[44] Donati P, Paolino G, Panetta C, Muscardin L, Cota C, et al (2013) Visceral leishmaniasis revealed by a squamous cell carcinoma in an HIV-1 infected patient. Infection 41: 575–578. 23378297 10.1007/s15010-013-0412-4

[45] Calza L, D'Antuono A, Marinacci G, Manfredi R, Colangeli V, et al (2004) Disseminated cutaneous leishmaniasis after visceral disease in a patient with AIDS. J Am Acad Dermatol 50: 461–465. 14988693 10.1016/j.jaad.2003.10.005

[46] Ramos A, Cruz I, Munez E, Salas C, Fernandez A, et al (2008) Post-kala-azar dermal Leishmaniasis and uveitis in an HIV-positive patient. Infection 36: 184–186. 18327683 10.1007/s15010-007-6279-5

[47] Kubar J, Marty P, Lelievre A, Quaranta JF, Staccini P, et al (1998) Visceral leishmaniosis in HIV-positive patients: primary infection, reactivation and latent infection. Impact of the CD4+ T-lymphocyte counts. AIDS 12: 2147–2153. 9833855 10.1097/00002030-199816000-00009

[48] Alvar J, Gutierrez-Solar B, Molina R, Lopez-Velez R, Garcia-Camacho A, et al (1992) Prevalence of Leishmania infection among AIDS patients. Lancet 339: 1427. 10.1016/0140-6736(92)91255-7 1350846

[49] Garcia-Garcia JA, Martin-Sanchez J, Gallego M, Rivero-Roman A, Camacho A, et al (2006) Use of noninvasive markers to detect Leishmania infection in asymptomatic human immunodeficiency virus-infected patients. J Clin Microbiol 44: 4455–4458. 17050814 10.1128/JCM.00921-06 PMC1698381

[50] Cota GF, de Sousa MR, Demarqui FN, Rabello A (2012) The diagnostic accuracy of serologic and molecular methods for detecting visceral leishmaniasis in HIV infected patients: meta-analysis. PLoS Negl Trop Dis 6: e1665. 22666514 10.1371/journal.pntd.0001665 PMC3362615

[51] ter Horst R, Tefera T, Assefa G, Ebrahim AZ, Davidson RN, et al (2009) Field evaluation of rK39 test and direct agglutination test for diagnosis of visceral leishmaniasis in a population with high prevalence of human immunodeficiency virus in Ethiopia. Am J Trop Med Hyg 80: 929–934. 19478251

[52] Monno R, Giannelli G, Rizzo C, De Vito D, Fumarola L (2009) Recombinant K39 immunochromatographic test for diagnosis of human leishmaniasis. Future Microbiol 4: 159–170. 19257843 10.2217/17460913.4.2.159

[53] World Health Organization (2011) Visceral leishmaniasis rapid diagnostic test performance. Diagnostic Evaluation Series No. 4. Geneva: World Health Organization. Available: http://www.who.int/tdr/publications/documents/vl-rdt-evaluation.pdf. Accessed 18 July 2014.

[54] Cruz I, Chicharro C, Nieto J, Bailo B, Canavate C, et al (2006) Comparison of new diagnostic tools for management of pediatric Mediterranean visceral leishmaniasis. J Clin Microbiol 44: 2343–2347. 16825347 10.1128/JCM.02297-05 PMC1489479

[55] Vilaplana C, Blanco S, Dominguez J, Gimenez M, Ausina V, et al (2004) Noninvasive method for diagnosis of visceral leishmaniasis by a latex agglutination test for detection of antigens in urine samples. J Clin Microbiol 42: 1853–1854. 15071070 10.1128/JCM.42.4.1853-1854.2004 PMC387579

[56] Riera C, Fisa R, Lopez P, Ribera E, Carrio J, et al (2004) Evaluation of a latex agglutination test (KAtex) for detection of *Leishmania* antigen in urine of patients with HIV-Leishmania coinfection: value in diagnosis and post-treatment follow-up. Eur J Clin Microbiol Infect Dis 23: 899–904. 15599651 10.1007/s10096-004-1249-7

[57] Bossolasco S, Gaiera G, Olchini D, Gulletta M, Martello L, et al (2003) Real-time PCR assay for clinical management of human immunodeficiency virus-infected patients with visceral leishmaniasis. J Clin Microbiol 41: 5080–5084. 14605142 10.1128/JCM.41.11.5080-5084.2003 PMC262523

[58] Mary C, Faraut F, Lascombe L, Dumon H (2004) Quantification of *Leishmania infantum* DNA by a real-time PCR assay with high sensitivity. J Clin Microbiol 42: 5249–5255. 15528722 10.1128/JCM.42.11.5249-5255.2004 PMC525214

[59] Cota GF, de Sousa MR, Rabello A (2011) Predictors of visceral leishmaniasis relapse in HIV-infected patients: a systematic review. PLoS Negl Trop Dis 5: e1153. 21666786 10.1371/journal.pntd.0001153 PMC3110161

[60] Moreno J, Canavate C, Chamizo C, Laguna F, Alvar J (2000) HIV–*Leishmania infantum* co-infection: humoral and cellular immune responses to the parasite after chemotherapy. Trans R Soc Trop Med Hyg 94: 328–332. 10975014 10.1016/s0035-9203(00)90345-6

[61] Bourgeois N, Lachaud L, Reynes J, Rouanet I, Mahamat A, et al (2008) Long-term monitoring of visceral leishmaniasis in patients with AIDS: relapse risk factors, value of polymerase chain reaction, and potential impact on secondary prophylaxis. J Acquir Immune Defic Syndr 48: 13–19. 18300698 10.1097/QAI.0b013e318166af5d

[62] Costa SR, D'Oliveira A Jr, Bacellar O, Carvalho EM (1999) T cell response of asymptomatic *Leishmania chagasi* infected subjects to recombinant leishmania antigens. Mem Inst Oswaldo Cruz 94: 367–370. 10.1590/s0074-02761999000300015 10348984

[63] Turgay N, Balcioglu IC, Toz SO, Ozbel Y, Jones SL (2010) Quantiferon-Leishmania as an epidemiological tool for evaluating the exposure to *Leishmania* infection. Am J Trop Med Hyg 83: 822–824. 20889872 10.4269/ajtmh.2010.09-0605 PMC2946749

[64] Gidwani K, Jones S, Kumar R, Boelaert M, Sundar S (2011) Interferon-gamma release assay (modified QuantiFERON) as a potential marker of infection for *Leishmania donovani*, a proof of concept study. PLoS Negl Trop Dis 5: e1042. 21526219 10.1371/journal.pntd.0001042 PMC3079582

[65] Alimohammadian MH, Jones SL, Darabi H, Riazirad F, Ajdary S, et al (2012) Assessment of interferon-gamma levels and leishmanin skin test results in persons recovered for leishmaniasis. Am J Trop Med Hyg 87: 70–75. 22764294 10.4269/ajtmh.2012.11-0479 PMC3391060

[66] Singh OP, Gidwani K, Kumar R, Nylen S, Jones SL, et al (2012) Reassessment of immune correlates in human visceral leishmaniasis as defined by cytokine release in whole blood. Clin Vaccine Immunol 19: 961–966. 22539471 10.1128/CVI.00143-12 PMC3370446

[67] Pintado V, Lopez-Velez R (2001) HIV-associated visceral leishmaniasis. Clin Microbiol Infect 7: 291–300. 11442562 10.1046/j.1198-743x.2001.00262.x

[68] van Griensven J, Carrillo E, Lopez-Velez R, Lynen L, Moreno J (2014) Leishmaniasis in immunosuppressed individuals. Clin Microbiol Infect 20: 286–299. 24450618 10.1111/1469-0691.12556

[69] Russo R, Nigro LC, Minniti S, Montineri A, Gradoni L, et al (1996) Visceral leishmaniasis in HIV infected patients: treatment with high dose liposomal amphotericin B (AmBisome). J Infect 32: 133–137. 8708370 10.1016/s0163-4453(96)91343-2

[70] Montalban C, Calleja JL, Erice A, Laguna F, Clotet B, et al (1990) Visceral leishmaniasis in patients infected with human immunodeficiency virus. Co-operative Group for the Study of Leishmaniasis in AIDS. J Infect 21: 261–270. 2273273 10.1016/0163-4453(90)93933-j

[71] Troya J, Casquero A, Refoyo E, Fernandez-Guerrero ML, Gorgolas M (2008) Long term failure of miltefosine in the treatment of refractory visceral leishmaniasis in AIDS patients. Scand J Infect Dis 40: 78–80. 17852921 10.1080/00365540701466215

[72] Rybniker J, Goede V, Mertens J, Ortmann M, Kulas W, et al (2010) Treatment of visceral leishmaniasis with intravenous pentamidine and oral fluconazole in an HIV-positive patient with chronic renal failure–a case report and brief review of the literature. Int J Infect Dis 14: e522–e525. 19726213 10.1016/j.ijid.2009.06.010

[73] Croft SL, Sundar S, Fairlamb AH (2006) Drug resistance in leishmaniasis. Clin Microbiol Rev 19: 111–126. 16418526 10.1128/CMR.19.1.111-126.2006 PMC1360270

[74] Collini P, Premchand N, Lockwood D, Greig J (2009) Successful use of miltefosine and sodium stibogluconate, in combination, for the treatment of an HIV-positive patient with visceral leishmaniasis: a case report and brief review of the literature. Ann Trop Med Parasitol 103: 455–459. 19583915 10.1179/136485909X451753

[75] Laguna F, Lopez-Velez R, Soriano V, Montilla P, Alvar J, et al (1994) Assessment of allopurinol plus meglumine antimoniate in the treatment of visceral leishmaniasis in patients infected with HIV. J Infect 28: 255–259. 8089514 10.1016/s0163-4453(94)91643-8

[76] Mastroianni A (2004) Liposomal amphotericin B and rHuGM-CSF for treatment of visceral leishmaniasis in AIDS. Infez Med 12: 197–204. 15711134

[77] Khan AR, Khan S, Zimmerman V, Baddour LM, Tleyjeh IM (2010) Quality and strength of evidence of the Infectious Diseases Society of America clinical practice guidelines. Clin Infect Dis 51: 1147–1156. 20946067 10.1086/656735

[78] Kish MA (2001) Infectious Diseases Society of America: Guide to development of practice guidelines. Clin Infect Dis 32: 851–854. 11247707 10.1086/319366

[79] Lopez-Velez R, Videla S, Marquez M, Boix V, Jimenez-Mejias ME, et al (2004) Amphotericin B lipid complex versus no treatment in the secondary prophylaxis of visceral leishmaniasis in HIV-infected patients. J Antimicrob Chemother 53: 540–543. 14739148 10.1093/jac/dkh084

[80] Molina I, Falco V, Crespo M, Riera C, Ribera E, et al (2007) Efficacy of liposomal amphotericin B for secondary prophylaxis of visceral leishmaniasis in HIV-infected patients. J Antimicrob Chemother 60: 837–842. 17684055 10.1093/jac/dkm294

[81] Ribera E, Ocana I, de Otero J, Cortes E, Gasser I, et al (1996) Prophylaxis of visceral leishmaniasis in human immunodeficiency virus-infected patients. Am J Med 100: 496–501. 8644760 10.1016/s0002-9343(97)89503-4

[82] Lopez-Velez R, Perez-Molina JA, Guerrero A, Baquero F, Villarrubia J, et al (1998) Clinicoepidemiologic characteristics, prognostic factors, and survival analysis of patients coinfected with human immunodeficiency virus and *Leishmania* in an area of Madrid, Spain. Am J Trop Med Hyg 58: 436–443. 9574788 10.4269/ajtmh.1998.58.436

[83] Patel TA, Lockwood DN (2009) Pentamidine as secondary prophylaxis for visceral leishmaniasis in the immunocompromised host: report of four cases. Trop Med Int Health 14: 1064–1070. 19552658 10.1111/j.1365-3156.2009.02329.x

[84] Perez-Molina JA, Lopez-Velez R, Montilla P, Guerrero A (1996) Pentamidine isethionate as secondary prophylaxis against visceral leishmaniasis in HIV-positive patients. AIDS 10: 237–238. 10.1097/00002030-199602000-00023 8838721

[85] Marques N, Sa R, Coelho F, Oliveira J, Saraiva Da Cunha J, et al (2008) Miltefosine for visceral leishmaniasis relapse treatment and secondary prophylaxis in HIV-infected patients. Scand J Infect Dis 40: 523–526. 18584541 10.1080/00365540701787800

[86] Angarano G, Maggi P, Coppola SL, Cavaliere RL (1998) Itraconazole as maintenance therapy for visceral leishmaniasis in HIV-infected patients. Eur J Clin Microbiol Infect Dis 17: 365–367. 9721971 10.1007/BF01709465

[87] Lafeuillade A, Chaffanjon P, Delbeke E, Quilichini R (1992) Maintenance itraconazole for visceral leishmaniasis in HIV infection. Am J Med 92: 449. 10.1016/0002-9343(92)90281-f 1313638

[88] Raffi F, Merrien D, Le Pape P, Reliquet V (1995) Use of an Itraconazole/allopurinol combination for the treatment of visceral leishmaniasis in a patient with AIDS. Clin Infect Dis 21: 1338–1339. 8589174 10.1093/clinids/21.5.1338

[89] Torrus D, Boix V, Massa B, Portilla J, Perez-Mateo M (1996) Fluconazole plus allopurinol in treatment of visceral leishmaniasis. J Antimicrob Chemother 37: 1042–1043. 8737162 10.1093/jac/37.5.1042

[90] Barragan P, Lopez-Velez R, Olmo M, Podzamczer D (2010) Visceral Leishmaniasis treated with antimonials/paromomycin followed by itraconazole/miltefosine after standard therapy failures in a human immunodeficiency virus-infected patient. Am J Trop Med Hyg 83: 10–12. 20595469 10.4269/ajtmh.2010.09-0594 PMC2912567

[91] Berenguer J, Cosin J, Miralles P, Lopez JC, Padilla B (2000) Discontinuation of secondary anti-leishmania prophylaxis in HIV-infected patients who have responded to highly active antiretroviral therapy. AIDS 14: 2946–2948. 11153679 10.1097/00002030-200012220-00020

[92] Soriano VF, Dona CF, Rodriguez-Rosado RF, Barreiro PF, González-Lahoz J (2000) Discontinuation of secondary prophylaxis for opportunistic infections in HIV-infected patients receiving highly active antiretroviral therapy. AIDS 14: 383–386. 10770540 10.1097/00002030-200003100-00011

[93] van Griensven J, Diro E, Lopez-Velez R, Boelaert M, Lynen L, et al (2013) HIV-1 protease inhibitors for treatment of visceral leishmaniasis in HIV-co-infected individuals. Lancet Infect Dis 13: 251–259. 23427890 10.1016/S1473-3099(12)70348-1

[94] Ridolfo AL, Gervasoni C, Antinori S, Pizzuto M, Santambrogio S, et al (2000) Post-kala-azar dermal leishmaniasis during highly active antiretroviral therapy in an AIDS patient infected with *Leishmania infantum* . J Infect 40: 199–202. 10841104 10.1053/jinf.1999.0630

[95] Antinori S, Longhi E, Bestetti G, Piolini R, Acquaviva V, et al (2007) Post-kala-azar dermal leishmaniasis as an immune reconstitution inflammatory syndrome in a patient with acquired immune deficiency syndrome. Br J Dermatol 157: 1032–1036. 17854365 10.1111/j.1365-2133.2007.08157.x

[96] Chicharro C, Llanes-Acevedo IP, Garcia E, Nieto J, Moreno J, et al (2013) Molecular typing of *Leishmania infantum* isolates from a leishmaniasis outbreak in Madrid, Spain, 2009 to 2012. Euro Surveill 18: 20545. 23929179 10.2807/1560-7917.es2013.18.30.20545

[97] Varani S, Cagarelli R, Melchionda F, Attard L, Salvadori C, et al (2013) Ongoing outbreak of visceral leishmaniasis in Bologna Province, Italy, November 2012 to May 2013. Euro Surveill 18: 20530. 23929116

[98] Gradoni L (2013) Epidemiological surveillance of leishmaniasis in the European Union: operational and research challenges. Euro Surveill 18: 20539. 23929176 10.2807/1560-7917.es2013.18.30.20539

[99] Hailu A, Berhe N (2002) The performance of direct agglutination tests (DAT) in the diagnosis of visceral leishmaniasis among Ethiopian patients with HIV co-infection. Ann Trop Med Parasitol 96: 25–30. 11989530 10.1179/000349802125000475

[100] Salvador F, Molina I, Sulleiro E, Burgos J, Curran A, et al (2013) Tropical diseases screening in immigrant patients with human immunodeficiency virus infection in Spain. Am J Trop Med Hyg 88: 1196–1202. 23509119 10.4269/ajtmh.12-0714 PMC3752822

